# Recent advances in *in situ* Notch signaling measurement

**DOI:** 10.3389/fcell.2023.1244105

**Published:** 2023-07-28

**Authors:** Masaharu Yoshihara, Satoru Takahashi

**Affiliations:** ^1^ Department of Primary Care and Medical Education, Institute of Medicine, University of Tsukuba, Tsukuba, Japan; ^2^ Department of Anatomy and Embryology, Institute of Medicine, University of Tsukuba, Tsukuba, Japan; ^3^ Transborder Medical Research Center, Institute of Medicine, University of Tsukuba, Tsukuba, Japan

**Keywords:** Cre/loxP system, Gal4/UAS system, lateral induction, lateral inhibition, patterning morphogenesis, transgenic mouse

## Abstract

Notch signaling is necessary for the development of many organ systems, including the nervous system, biliary system, and visual and auditory sensory systems. This signaling pathway is composed of DSL ligands and Notch receptors. Upon the interaction of those components between neighboring cells, the intracellular domain of the Notch receptor is cleaved from the cell membrane to act as a transcription factor. To date, many mechanistic insights, including lateral inhibition and lateral induction, have been proposed from observation of patterning morphogenesis and expression profiles of Notch signaling-associated molecules. The lack of a direct measurement method for Notch signaling, however, has impeded the examination of those mechanistic insights. In this mini-review, recent advances in the direct measurement of Notch signaling are introduced with a focus on the application of genetic modification of Notch receptors with the components of the Cre/loxP system and Gal4/UAS system. The combination of such conventional genetic techniques is opening a new era in Notch signaling biology by direct visualization of Notch “signaling” in addition to Notch signaling-associated molecules.

## Introduction

### Biochemistry of Notch signaling

The Notch signaling pathway is a well-conserved signaling pathway (King et al., 2008; Gazave et al., 2009) that is composed of the DSL (Delta/Serrate/Lag2) ligands and Notch receptors (Kopan and Ilagan, 2009). From a biochemical point of view, upon the binding of the DSL ligands and Notch receptors, the intracellular domain of Notch receptors (NICD) is cleaved from the cell membrane and translocated in the nucleus to transduce the signal (Struhl and Adachi, 1998). The Notch downstream target genes include Hes1, Hes5, and Hey1. However, this signaling is far more complex than this due to the many associated enzymes in glycosylation-mediated (reviewed by Pandey et al., 2021) or ubiquitin-mediated (reviewed by Bray, 2006) Notch trafficking and phosphorylation-mediated NICD turnover (Fryer et al., 2004). In addition, Notch receptor subtype (Notch 1–4)-specific examination is ideal because the contributions of Notch1 and Notch2 in the kidney development, for example, are different (Cheng et al., 2007) that was partially owing to the difference in the membrane trafficking and cleavage efficiency between Notch1 and Notch2 (Liu et al., 2013). As a result, the direct measurement of Notch signaling itself rather than observation of Notch signaling-associated molecules is the focus of this review.

**FIGURE 1:**
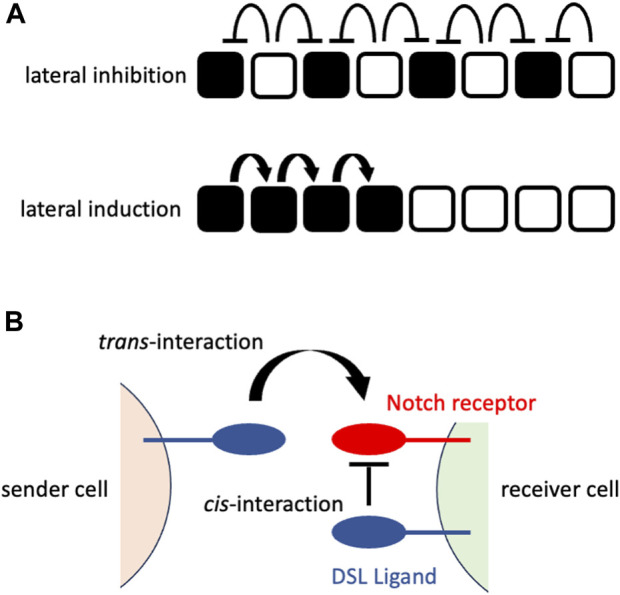
Notch signaling at cellular or molecular levels **(A)** Notch signaling at a cellular level. The upper panel shows the consequence of lateral inhibition. The DSL ligand-expressing cells (white cells) inhibit the expression of the Notch receptors in their neighboring cells (signaling-receiving cells; black cells), resulting in the fine-grained pattern. The lower panel shows the consequence of lateral induction. The signaling-receiving cells (black cells) sends Notch signaling to their neighboring cells, resulting in the spatial expansion of signaling-receiving cells. The white cells represent the cells that have not received Notch signaling. **(B)** Notch signaling at a molecular level. The sender cell expresses the DSL ligand on its cell surface that interacts with the Notch receptor on the cell surface of the neighboring (receiver) cell (trans-interaction). This interaction transduces Notch signaling in the receiver cells. By contrast, the interaction of the DSL ligand and Notch receptor on the same cell surface fastens the patterning morphogenesis rather than transducing the Notch signaling.

The early mechanistic concepts of patterning morphogenesis via the Notch signaling pathway came from the observation of *Drosophila* neurogenesis, where Notch signaling dictates undifferentiated ectodermal cells to differentiate into neural cells that are located in a scattered fashion in epidermal cells (Artavanis-Tsakonas et al., 1983; Goriely et al., 1991; Heitzler and Simpson, 1991). The mechanism behind the generation of this “salt-and-pepper” or “fine-grained” pattern from an initially homogenous undifferentiated cell population was mathematically formulated and named “lateral inhibition with feedback” (Collier et al., 1996). In lateral inhibition with feedback, Notch signaling-receiving cells express fewer DSL ligands that gradually augment the initial fluctuation of Notch signaling among the cells to generate a “salt-and-pepper” pattern (Figure 1A). In addition to such *trans*-interaction of DSL ligands and Notch receptors between neighboring cells, a recent biochemical study suggested the presence of *cis*-interaction of those ligands and receptors within the same cell membrane (Sprinzak et al., 2010) that shortens the time to reach a “salt-and-pepper” pattern (Sprinzak et al., 2011) (Figure 1B).

### Patterning morphogenesis via Notch signaling

Notch signaling orchestrates patterning morphogenesis along with determining cell fates in many developmental contexts, including intrahepatic bile duct (IHBD) formation (Sparks et al., 2010), retina (Ahmed et al., 1995) and inner ear (Haddon et al., 1998). There are histological gaps with each other that should be considered when Notch signaling-mediated patterning morphogenesis is formulated.

IHBD is formed from the ductal plate that is a one- or two-cell layer surrounding the portal vein in the liver (reviewed by Roskams and Desmet, 2008). The smooth muscle cells of the portal vein express one of the DSL ligands (Jagged1) to induce the differentiation of ductal plate cells from undifferentiated hepatoblasts via Notch2 signaling (Tanimuzu and Miyajima, 2004; Hofmann et al., 2010). Indeed, Jagged1 (Li et al., 1997; Oda et al., 1997) and Notch2 (McDaniell et al., 2006) are responsible for Alagille syndrome, which causes malformation of IHBD in humans. Importantly, ductal plate cell features (expression of cytokeratins) are spatially limited to the one- or two-cell layer in wild-type mice, while Jagged1 knockout in portal smooth muscle cells led to widespread expression of cytokeratins and subsequent loss of IHBD formation (Hofmann et al., 2010). Therefore, Notch signaling converts the spatial signature to a molecular signature in IHBD formation, whereas Notch signaling defines the spatial signature in *Drosophila* neurogenesis. A recent computer simulation study suggested that ductal plate formation (spatially confined patterning) was achieved under some special DSL ligand or Notch receptor production rates (production rates of either DSL ligand or Notch receptors were low in hepatoblasts) otherwise achieving fine-grained patterning (Yoshihara et al., 2021). In this situation, spatially confined patterning was achieved owing to the interaction of DSL ligands and Notch receptors that occurs almost exclusively between the portal smooth muscle cells and their neighboring cells. Experimental examination of that simulation result, however, was lacking owing to a technical difficulty in measurement of Notch signaling at a cellular level *in vivo*.

The retina is a neural tissue in the eyeball that has three neuronal cell layers (ganglion cell layer, inner nuclear layer and outer nuclear layer) and interspersed glial cells. During their development, ganglion cells are first generated from multipotent retinal progenitor cells and other types of cells, and those layers are subsequently generated (reviewed by Bassett and Wallace, 2012). In the rat retina, Notch1 expression was widely observed at embryonic Day 14 (E14) and subsequently confined to the ganglion cell layer at E16 (Ahmed et al., 1995). A recent study suggested that retinal pigment epithelium (the cell layer outer to ganglion cell layer) expresses DSL ligands at its apical surface to maintain neuronal progenitor cells that can undergo asymmetrical division at mouse E14.5 (Ha et al., 2017). The same study also suggested that retinal progenitor cells express three DSL ligands (Dll1, Dll3, and Dll4) and two Notch receptors (Notch1 and Notch2), while Mib1-mediated apical surface translocation of DSL ligands in the retinal pigment epithelium is important to Notch signaling in retinal progenitor cells. Therefore, in contrast to the IHBD, the retina is an example where Notch signaling above the threshold level was spatially confined to neuronal progenitor cells other than their daughter cells, although the expression of DSL ligands and Notch receptors was widespread. Direct measurement of Notch signaling is, however, necessary to examine the patterning mechanism because Notch downstream target genes (Hes1, for example,) are induced by multiple signaling pathways, such as the Wnt signaling pathway and Hedgehog signaling pathway (reviewed by Rani et al., 2016).

Inner ear development is an example of another mode of operation of Notch signaling called lateral induction (Figure 1A) (Brown II et al., 2020). In contrast to lateral inhibition, lateral induction that is mediated by a positive feedback loop for DSL ligands generates homogenous cell states among neighbors (reviewed by Bocci et al., 2020). The opposing phenomena (lateral inhibition and lateral induction) take place in the developing inner ear (Eddison et al., 2000). Using chicken embryos, it has been proposed that Jagged1 expression is associated with Hey1 expression in a manner resembling lateral induction, while Delta 1 (another DSL ligand) antagonizes Jagged1 to elicit lower Notch signaling, which results in Hey1/Hes5 expression in a manner resembling lateral inhibition (Petrovic et al., 2014). The important point here, however, is that the progression pattern of Notch signaling, as measured by its downstream target gene expression, is clearly different from other biological contexts, such as *Drosophila* neurogenesis, IHBD formation and retina formation. To examine whether such spatial progression of differentiation is indeed dictated by Notch signaling, it is necessary to directly measure that signaling *in situ*, hopefully distinguishing past and ongoing signaling.

### Measurement of Notch signaling

Notch signaling dynamics have been examined in multiple ways (reviewed by Gridley and Groves, 2014). For example, *in situ* hybridization and immunofluorescence for Notch receptors or their downstream target genes were used in the early days (for example, Ahmed et al., 1995). Among these methods, the presence of anti-NICD antibodies in the nucleus was strong evidence of ongoing activation of Notch receptors (for example, Cheng et al., 2007; Salguero-Jiménez et al., 2018). These methods mark only ongoing Notch signaling but neither past Notch signaling nor whole picture of contribution of Notch signaling, and potentially have technical problems in *in situ* hybridization and immunofluorescence although there are successful cases as mentioned above. Genetics also enabled visualization of ongoing Notch activity with tag or fluorescent proteins. For example, the emerald variant of GFP was expressed under the regulation of the Hes1 promotor (Fre et al., 2011). Alternatively, nls (nucleus localization signal)-lacZ was expressed under the regulation of Rbpj (common mediator of Notch signaling) binding sites (Souihol et al., 2006). In another study, a yellow fluorescent protein, Venus, was expressed under the regulation of Cbf1 (Cbf1 is an alternative name for Rbpj) binding sites (Mizutani et al., 2007). These methods, however, failed to distinguish the subtype of Notch receptors upstream of the reporter signaling.

**FIGURE 2:**
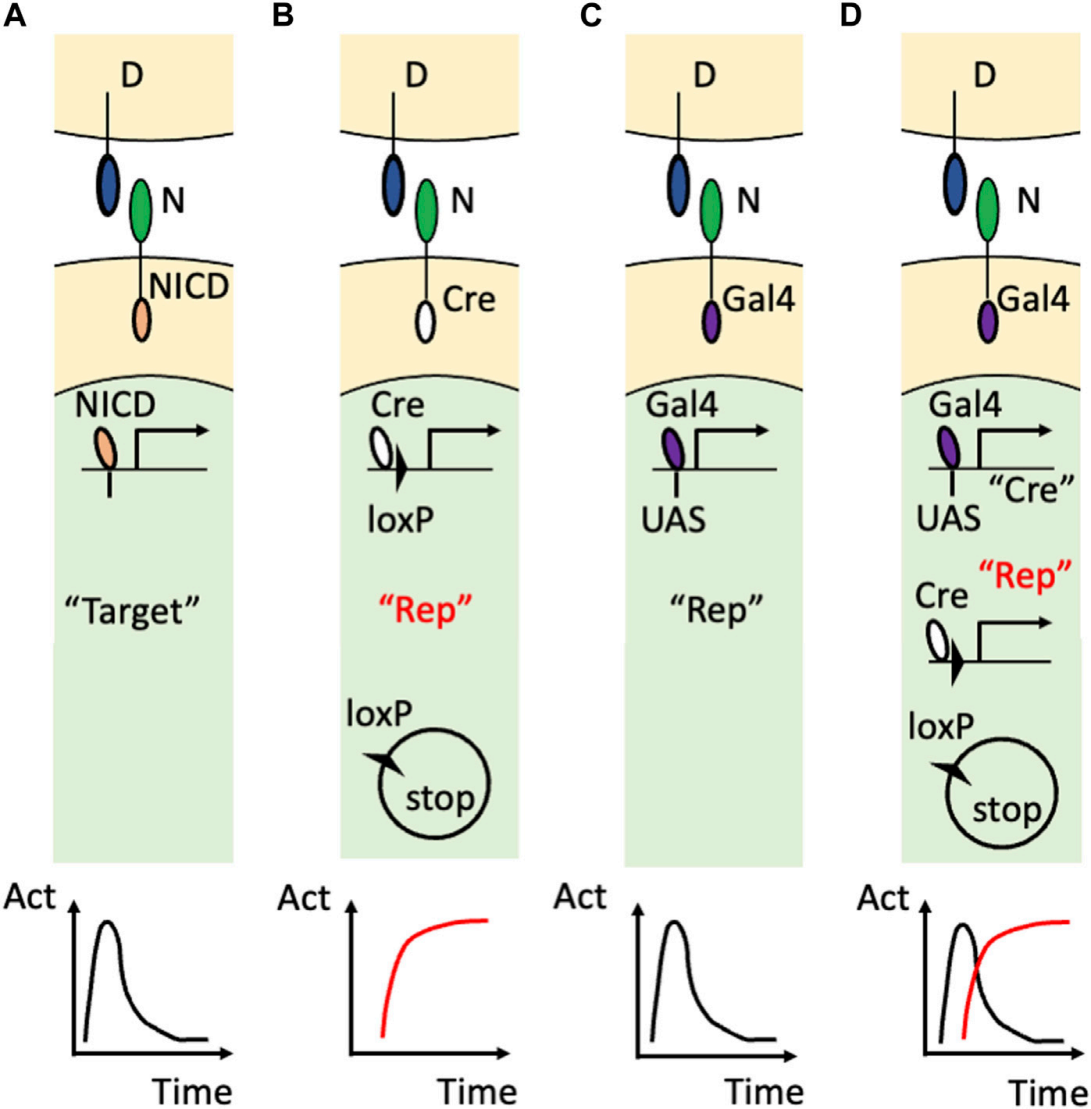
Visualization of Notch signaling in transgenic mice **(A)** The action of the endogenous Notch signaling. The binding of the DSL ligand (“D”) and Notch receptor (“N”) induces translocation of the NICD into the nucleus to induce the expression of the endogenous target genes (“Target”). Note that this target gene expression is temporal as shown in the time-activity (“Act”) graph at the bottom. **(B)** The utilization of the Cre/loxP system. The endogenous NICD is replaced with Cre recombinase that removes the floxed stop sequence and allows the permanent reporter expression (“Rep”). The permanent expression of the reporter is the past Notch signaling and is highlighted in red. **(C)** The utilization of the Gal4/UAS system. The endogenous NICD is replaced with Gal4VP16 that binds to the transgenic UAS sequence to induce the temporal expression of the reporter. **(D)** Combining the Gal4/UAS system and Cre/loxP system. The endogenous NICD is replaced with Gal4VP16 that binds to the transgenic UAS sequence to induce the temporal expression of Cre recombinase. Cre recombinase allows the permanent expression of the reporter in addition to being used as the marker of the temporal activation of the Notch receptor. Note that the ongoing (temporal) signaling is highlighted with the double quotation (“Cre”) whereas the past (permanent) signaling is highlighted with the red color and double quotation (“Rep”).

The innovation came from using artificial Notch receptors. Endogenous Notch receptors undergo cleavage upon binding to their ligands to release their own NICD (Figure 2A). A research group led by Raphael Kopan replaced the NICD of the endogenous Notch1 receptor with Cre recombinase (Vooijs et al., 2007) and developed an improved version of that transgenic mouse by modulating the carboxy terminus of Cre recombinase and the polyadenylation signal (Liu et al., 2015) (Figure 2B). In these transgenic mouse lines, once the Cre-fused Notch1 receptor binds to endogenous DSL ligands, Cre recombinase is released from the cell membrane and acts in the nucleus. Concretely, the cleaved and translocated Cre recombinase removes the stop cassette between the loxP sequences and allows the expression of the reporter genes while Cre recombinase itself would undergo degradation. Therefore, past Notch1 signaling was marked in combination with other reporter mice, such as Rosa lacZ and Rosa EYFP, although ongoing Notch activity was missing in that reporter system. For example, in the kidney, this technology enabled the permanent labeling of the glomerular endothelial cells and tubular epithelial cells. Another research group led by Freddy Radtke fused an artificial transcription factor (Gal4VP16) with the Notch1 receptor (Notch1-Gal4VP16) and visualized ongoing Notch1 signaling in combination with UAS-lacZ reporter mice (Figure 2C) (Smith et al., 2012). For example, this technology showed the spatially restricted Notch1 signaling at the intestinal crypt cells. Past Notch activity, however, was missed in that reporter system.

Recently, the combination of the Gal4/UAS system and the Cre/loxP system enabled visualization of both past and ongoing Notch1 activity (Figure 2D) (Yoshihara et al., 2022). In this study, Cre recombinase was connected with a near infrared fluorescent protein, miRFP670 (Shemetov et al., 2017), by the Thosea asigna virus 2A (T2A) self-cleavage peptide and was expressed under the regulation of Gal4VP16 owing to the upstream activation sequence (UAS) (UAS-Cre-T2A-miRFP670 mouse). The ongoing Notch1 signaling was marked with Cre recombinase or miRFP670 in Notch1-Gal4VP16; UAS-Cre-T2A-miRFP670 mice. By using another reporter mouse that allowed expression of EGFP or tandem DsRed before and after Cre-mediated recombination (R26GRR mouse) (Hasegawa et al., 2013), past Notch1 signaling was also visualized with tandem DsRed at the cellular level. Indeed, both past and ongoing Notch1 signaling was visualized in the retina (Yoshihara et al., 2022) and kidney (Sugiura et al., 2023). Concretely, most of the embryonic day 14.5 retinal cells were positive for the Cre expression (ongoing Notch1 signaling) and the retinal cells at an adult stage were positive for the tandem DsRed expression (past Notch1 signaling) (Yoshihara et al., 2022). This technology also revealed temporal activation of Notch1 signaling because the renal tubular epithelial cells that received Notch1 signaling during their development (Liu et al., 2015) were largely negative for the Cre expression (Sugiura et al., 2023)

## Discussion

Measurement and perturbation are the major methods to examine the mechanisms behind phenomena. Concerning patterning morphogenesis via the Notch signaling pathway, visualization of Notch signaling *in situ* has been attempted for decades (for example, Ahmed et al., 1995; Vooijs et al., 2007; Smith et al., 2012). However, there are two major obstacles in clarifying the mechanism of patterning morphogenesis. The first obstacle comes from the fact that Notch signaling is a ligand‒receptor signaling pathway. The Notch signal transduction requires not only expression of both DSL ligands and Notch receptors but also the interactions between those ligands and receptors, which is subject to anatomical (positional) restrictions. Faced with technical difficulties in direct measurement of the interaction of DSL ligands and Notch receptors, downstream target gene expression was measured instead, although it is also induced by signaling pathways other than the Notch signaling pathway (reviewed by Rani et al., 2016). To measure ongoing signaling, immunohistochemical methods have been employed to show the NICD in the nucleus (for example, Cheng et al., 2007; Salguero-Jiménez et al., 2018). However, utilization of the Cre/loxP system (Vooijs et al., 2007; Liu et al., 2015) or Gal4/UAS system (Smith et al., 2012) overcame the obstacle of the ligand‒receptor signaling pathway without special techniques in *in situ* hybridization and immunofluorescence. The second obstacle comes from the technical difficulty in simultaneous monitoring of both past and ongoing signaling. Fusing Cre/loxP components to the Notch1 receptor enabled past Notch1 signaling to be marked (Vooijs et al., 2007; Liu et al., 2015), whereas fusing Gal4/UAS components to the Notch1 receptor enabled ongoing Notch1 signaling to be marked. Both methods were innovative, although owing to the lack of simultaneous labeling of past and ongoing Notch1 signaling, it is difficult to show the whole picture and stage-specific contribution of Notch1 signaling. The combination of the Gal4/UAS system and the Cre/loxP system resolved this obstacle (Yoshihara et al., 2022; Sugiura et al., 2023).

The turnover rates of the endogenous NICD and the reporters in the Gal4VP16 system (including Cre recombinase in the Gal4/UAS; Cre/loxP-combined system) would affect the visualization of ongoing Notch1 signaling. For example, on one hand, the intestinal crypt cells were labeled with ongoing Notch1 signaling in the Gal4/UAS system (Smith et al., 2012). On the other hand, those cells were not labeled with ongoing Notch1 signaling in the Gal4/UAS; Cre/loxP-combined system (Yoshihara et al., 2022). Since the latter system showed past Notch1 signaling in the intestinal epithelial cells, the observation using the former system is reasonable. This discrepancy might originate from the difference in the turnover rates between the reporters. Therefore, careful consideration on the reporters is necessary when ongoing Notch1 signaling is examined. To our best knowledge, unfortunately, detailed documentation of the exact turnover rates of the reporters in a specific cell type is lacking.

Recently, the Notch receptor cleavage has attracted attention. A research group led by Wendell Lim explored the use of the surface-tethered GFP as a ligand by utilizing anti-GFP binding nanobody and Gal4VP64 or TetRVP64 that are fused with the mouse Notch1 minimal regulatory region (Ile1427 to Arg1752 corresponding to the cleavage site) (they named this synthetic Notch receptor as “synNotch”) (Morsut et al., 2016). They expanded the synNotch system to study diffusible morphogen systems by introducing morphogen-anchoring cells to their original synNotch system (Toda et al., 2020). Another group led by Bin Zhou applied the synNotch system to living mice to trace the cell‒cell contact history *in vivo* (Zhang et al., 2022). Integration of the synNotch system and Gal4/UAS; Cre/loxP-combined system would enable labeling of both the past and ongoing signature of molecular interaction and cell-cell contact *in vivo*. Despite these innovative applications of Notch receptors to developmental biology, the monitoring of both past and ongoing endogenous Notch signaling has long been missing. Combining the Gal4/UAS system and the Cre/loxP system is a simple but promising method in Notch signaling biology. Now that a part of that system (UAS-Cre-T2A-miRFP670 mouse and R26GRR) has been developed (Hasegawa et al., 2013; Yoshihara et al., 2022), just fusing Gal4VP16 to the specific endogenous Notch receptors (Notch2, Notch3, and Notch4) could be enough to expand measuring past and ongoing Notch signaling to other Notch receptor subtypes.

Finally, some transgenic mouse resources, including UAS-Cre-T2A-miRFP670 mice (RIKEN BRC Number: RBRC11716) and R26GRR mice (RIKEN BRC Number: RBRC04874), are available from the National BioResource Project of Japan through the Institute of Physical and Chemical Research (RIKEN). Although past studies have provided many mechanistic insights, Notch signaling biology in patterning morphogenesis is still ongoing.
